# Effectiveness of an Integrated Mobile Application for Lifestyle Modifications in Overweight Women with Polycystic Ovarian Syndrome: A Randomized Controlled Trial

**DOI:** 10.3390/life13071533

**Published:** 2023-07-10

**Authors:** Haneul Lee, Seon-Heui Lee

**Affiliations:** 1 Department of Physical Therapy, College of Health Science, Gachon University, Incheon 21936, Republic of Korea; leehaneul84@gachon.ac.kr; 2 Department of Nursing, College of Nursing, Gachon University, Incheon 21936, Republic of Korea

**Keywords:** lifestyle modification, mobile application, polycystic ovary syndrome, weight loss

## Abstract

Polycystic ovary syndrome (PCOS) is a common endocrine disorder in women, characterized by hormonal imbalances and metabolic disturbances. Lifestyle modifications, including weight loss, are crucial for the management of PCOS symptoms. Mobile applications have emerged as promising tools to support lifestyle modifications. This study aimed to evaluate the effectiveness of a 12-week integrated lifestyle modification program, which used a mobile application, among overweight women with PCOS. A randomized controlled trial was conducted with 28 participants, who were assigned to either treatment group, which received a 12-week lifestyle modification program facilitated by a mobile application, or the control group, who were instructed to maintain their usual lifestyle and received an evidence-based leaflet containing information on PCOS. The primary outcome measure was a change in weight over 12 weeks. Insulin resistance, levels of sex hormones, hirsutism, acne, and depressive symptoms were measured as secondary outcomes. The results showed significant weight loss in the treatment group compared to the control group (3.19 vs. 0.79 kg; *p* < 0.05). Similarly, significant improvements were observed across time in postprandial insulin levels (22.25 vs. 9.29 μIU/mL), hirsutism (2.98 vs. −0.16 points), and depression (7.73 vs. 1.7 points) (*p* < 0.05) between the two groups. This study highlights the potential benefits of using a mobile application to support lifestyle modifications, including weight loss and improvement in depressive symptoms, in overweight women with PCOS. Further research is needed to explore the long-term effects and incorporation of advanced technologies to enhance PCOS management outcomes. Mobile applications for lifestyle modifications offer a promising avenue for addressing the unique challenges faced by women with PCOS and provide accessible and personalized support for their health needs.

## 1. Introduction

Polycystic ovary syndrome (PCOS) is a complex condition that affects various aspects of women’s health, including the reproductive and endocrine systems. It is prevalent among women of childbearing age, affecting approximately 8–13% of reproductive-aged women [1]. The diagnostic standard for PCOS, known as the Rotterdam criteria, requires the presence of at least two of the following three conditions: oligo/anovulation, hyperandrogenism, and polycystic ovaries on ultrasonography [2]. PCOS symptoms have a significant impact on the quality of life (QoL) of women, causing stress and negatively affecting their psychological well-being and sexuality [3]. Women with PCOS are at an increased risk of depression, anxiety, and personality disorders [4]. Therefore, addressing both the physical and emotional aspect of PCOS is crucial for its effective management.

Obesity is a major risk factor for the development of PCOS and can exacerbate its symptoms. The rising prevalence of PCOS in young women and the associated reproductive and metabolic risks of obesity have significant clinical and public health implications [5]. The prevalence of PCOS is higher in women with a body mass index (BMI) > 30 kg/m^2^ than in those with a BMI of 25 kg/m^2^ or lower [5]. Obesity can lead to irregular menstrual cycles, insulin resistance, hyperinsulinemia, hirsutism, hyperandrogenism, and nonovulatory menstruation. There is a risk of negative pregnancy outcomes and complications in women with PCOS [6]. In addition, PCOS is associated with a higher prevalence of hyperlipidemia, mental health issues, and sexual dysfunction [7,8,9,10,11].

The International Evidence-Based Guidelines for the Assessment and Management of PCOS 2018 recommend three main treatment options for PCOS: lifestyle intervention, pharmacological treatment, and bariatric surgery. However, pharmacological treatment has limitations such as side effects due to combined oral contraceptive pills like nausea, headache, dizziness, and spotting [12], and significantly increases the risk of venous thromboembolism [13]. Bariatric surgery is recommended only as a last resort when other weight loss methods fail due to its complexity [14]. Therefore, the guidelines prioritize lifestyle modifications, including dietary changes, increased physical activity, and behavioral interventions, as top priorities in the management of PCOS [15].

While traditional lifestyle modifications have been effective [16], face-to-face intervention programs have encountered a dropout rate as high as 62%, often because of time constraints [17,18]. Mobile health applications offer accessibility and affordability, allowing for users to engage in health management anytime and anywhere [19]. Studies conducted on various mobile health applications for women with PCOS have demonstrated cost-saving benefits and reduced hospital visits [20,21]. However, many existing PCOS-related applications fail to address patient needs adequately, lack comprehensive features, or provide insufficient quality information [22]. To address these gaps, we have developed an integrated mobile application that aims to motivate lifestyle modifications, manage the menstrual cycle, and provide evidence-based information regarding diet, exercise, disease, and its management [23]. By synthesizing the available evidence from systematic reviews and based on the assessments of the needs of women with PCOS [16], our application seeks to enhance the effectiveness of PCOS treatment and empower women with PCOS to actively manage their condition.

The primary objective of this study was to analyze the effectiveness of a 12-week integrated mobile application for lifestyle modifications in overweight women with PCOS. We hypothesized that this application would reduce obesity and improve overall health outcomes.

## 2. Materials and Methods

### 2.1. Study Design and Ethical Approval

This randomized controlled trial analyzed the effectiveness of a 12-week lifestyle modification mobile application in overweight patients with PCOS. It was conducted in accordance with the Consolidated Standards of Reporting Trial (CONSORT) recommendations [24] and registered on the cirs.nih.go.kr website (KCT0006062). This study was approved by the Institutional Review Board (IRB) of Gil Medical Center (GBIRB 2021-065) and was conducted in accordance with the Declaration of Helsinki. All participants were provided with a detailed explanation of the purpose of the study and privacy policy before beginning the trial. Informed consent was obtained from all participants who agreed to participate in the study.

### 2.2. Participants

A total of 34 women aged 18–40 years, who were diagnosed with PCOS based on the Rotterdam criteria [25], had a BMI greater than 25 kg/m^2^, and reported menstrual irregularities were recruited from the outpatient department of Obstetrics and Gynecology at Gil Medical Center from October 2021 to December 2022. Women who were pregnant with endocrine disorders, those taking insulin resistance-reducing drugs, and those with a history of eating disorders or significant weight change in the past year were excluded from the study.

**Figure 1:**
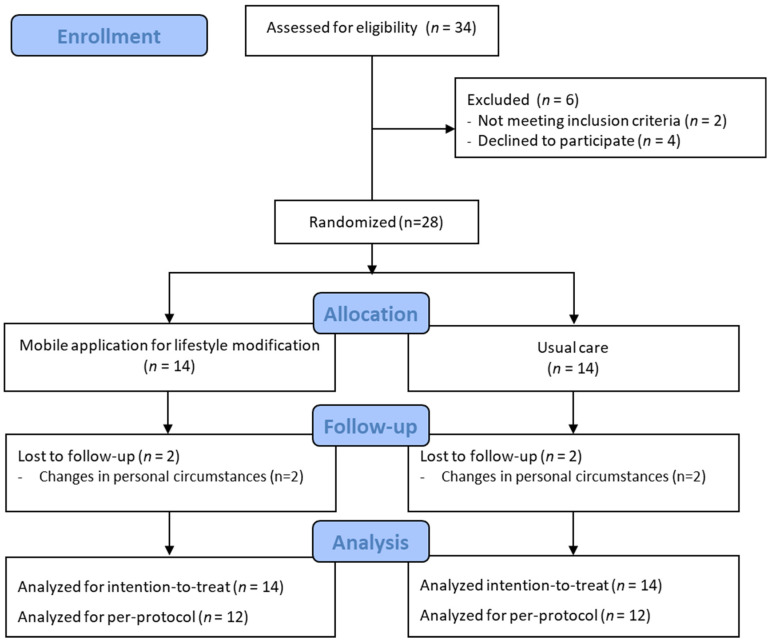
Flow diagram of the study.

Of the 34 patients, 4 declined to participate in the study and 2 did not meet the inclusion criteria. Finally, 28 women with PCOS were included in this study. They were randomly assigned to either the treatment group (lifestyle modification app users) or the control group (usual care) (Figure 1).

### 2.3. Randomization and Blinding

Randomization was conducted by the research coordinator, who independently performed simple randomization using an online software called ‘research randomizer’ before data collection. The software randomly assigned 14 numbers from 1 to 28 to the treatment and control groups. Subsequently, numbers were assigned to the participants based on the order of recruitment. This ensured that all study investigators assessed the outcome variables and that the participants were unaware of their group assignments until the completion of the analysis.

### 2.4. Intervention

**Figure 2:**
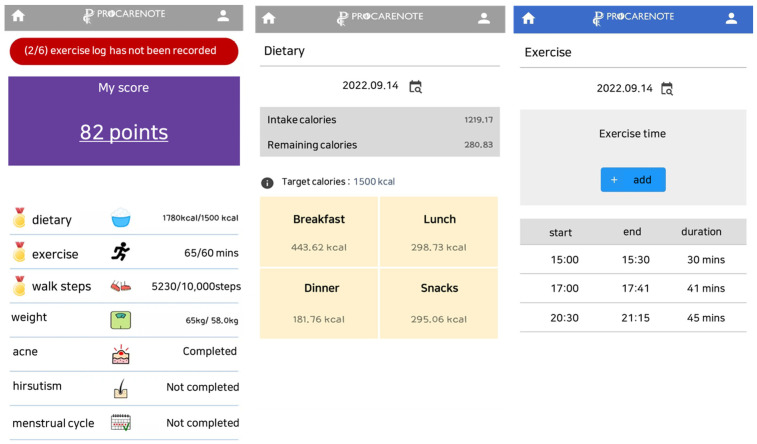
Integrated mobile application for lifestyle modifications.

The treatment group participated in a 12-week lifestyle modification program facilitated by a mobile application developed by our research team [23]. Upon registration, the participants were provided with an evidence-based booklet containing comprehensive information about PCOS, including details about the disease, symptoms, diet, and exercise (Figure 2). Throughout the 12 weeks, participants were required to enter various details daily into the mobile application, including intake amount, exercise duration, daily body weight, and gynecological information such as menstrual cycles, pregnancy, and other data. The application automatically calculated the total calories, exercise time, hirsutism, and acne scale scores based on the recorded data. The application was designed to enable users to complete questionnaires, access educational resources, and communicate with researchers for counseling. To motivate participation, users were provided with scores based on their progress towards their daily goals. The researcher reviewed these records and contacted each participant twice a week through mobile feedback messages or phone calls. The feedback aimed to assist participants in achieving their target exercise time of an hour (or 10,000 steps of walk) with moderate intensity and maintaining their caloric intake between 1400 and 1500 kcal/day. The prescribed macronutrient composition of the diet consisted of approximately 20% of total energy intake from protein, 30% from fat, and 50% from carbohydrates [26]. Emotional health was monitored using app notifications or text messages. Participants who successfully achieved their goals received positive feedback, whereas those who faced challenges were provided with feedback to encourage and motivate them. Furthermore, monthly phone calls or visits to the research center were conducted to address any difficulties in using the application or following the lifestyle modification program.

The control group was instructed to maintain their usual lifestyle and received an evidence-based leaflet containing information about PCOS at the beginning of the study.

### 2.5. Outcome Measures

The primary outcome of this study was a change in weight while using a mobile application for PCOS for 12 weeks.

The secondary outcomes included insulin resistance, hormone levels, productivity indicators, hirsutism, acne scores, and emotional state. Blood samples were collected from the participants to analyze the levels of luteinizing hormone (LH), follicle-stimulating hormone (FSH), estradiol, testosterone, sex hormone-binding globulin (SHBG), and dehydroepiandrosterone sulfate (DHEA-S). The research nurses collected 3 to 5 mL of blood from the antecubital vein using a 23-gauge needle in separate serum tubes. To maintain sample integrity, the blood samples were promptly dispatched to the laboratory on the day of collection. In the laboratory setting, data derived from blood samples were meticulously analyzed following the precise guidelines provided by the manufacturer. LH, FSH, E2, SHBG, DHEA-S, and insulin levels were quantified using Elcsys assay kits (Roche Diagnostics, Meylan, France), and glucose levels were estimated using Glucose HK Gen 3 (Roche Diagnostics, Meylan, France).

Hirsutism was assessed using the Ferriman–Gallwey score. This scoring system ranges from 0 to 36, with higher scores indicating more extensive hair growth [27]. Acne was assessed using the Global Acne Grading System (GAGS). The score ranges from 0 to 52, and a higher GAGS score indicates more severe acne [28]. The Korean version of the Epidemiological Studies Depression Scale (K-CESD) was used to evaluate depressive symptoms. The scale is scored from 0 to 60, with higher scores indicating depressive symptoms of greater severity [29]. Furthermore, the participants’ menstrual irregularities and pregnancy status were carefully monitored throughout the study.

For women who did not experience amenorrhea, blood samples were obtained during days 2–7 of their menstrual cycle, before and 12 weeks after the intervention. For women who did not have regular menstrual cycles, blood was collected within one week after the trial began, before the intervention, and 12 weeks after the intervention. Other outcomes were assessed based on their respective blood collection schedules.

### 2.6. Sample Sizes

The sample size of this study was calculated using the G Power 3.1.9.7 software (Heinrich Heine University, Dusseldorf, Germany). A previous study reported a moderate effect of weight loss (*d* = 0.593) [30]. However, no previous study reported the effect size for the time × group interaction in weight loss; thus, a medium effect size of 0.3 was considered to calculate the sample size with a significance level of 0.05 and a power of 80%. Twenty-four participants were required to detect statistical significance when a clinically significant interaction was observed between time points and groups. An additional 25% were recruited to prepare for an unexpected elimination.

### 2.7. Statistical Analysis

Statistical analyses were performed based on an intent-to-treat principle using the IBM SPSS software (version 26.0; IBM, Armonk, NY, USA). The last observation carried forward method was used for patients who completed at least one set of data measurements. Data are summarized as means and standard deviation (SD). The normality of the distributions was tested using the Shapiro–Wilk test and an independent *t*-test was performed to compare the general characteristics and baseline data between the groups. For non-parametric statistics, the Generalized Estimated Equation (GEE) for repeated-measures analysis was used to examine changes in outcomes over time after adjusting for hirsutism and depression scales, as their baseline characteristics significantly differed between the groups. The GEE method included interaction terms between groups (intervention vs. control) and time (pre-test vs. post-test) [31]. The effect size was estimated using Carmer’s phi (φ_c_), where a value of 0.1 was interpreted as a small effect size, 0.3 as medium, and 0.5 as a large effect size [32]. The level of statistical significance was set at 0.05.

## 3. Result

Of the 28 participants, 4 participants (2 each in the treatment and control groups) dropped out of the study because of schedule conflicts. Therefore, 24 participants were included in this study. There were no significant differences in the general characteristics and outcome measures between participants who dropped out and those who completed the study. Except for the hirsutism score (14.83 ± 2.66 vs. 14.83 ± 2.66, *p* = 0.015) and depressive symptoms (24.15 ± 7.03 vs. 16.08 ± 7.63, *p* = 0.039), the baseline characteristics were similar between the two groups, as shown in Table 1. Of the 28 patients, the mean age was 26.46 ± 4.41 years, the mean body weight was 73.15 ± 9.47 kg, and the mean BMI was 28.18 ± 4.98 kg/m^2^. The baseline characteristics of the participants are presented in Table 1.

There was a significant difference in weight loss between the two groups over time after adjusting for hirsutism and depression scores (*X*^2^ = 3.94, *p* = 0.042, φ_c_ = 0.37). The treatment group showed a 4.4% weight loss (from 75.84 to 72.65 kg) after the 12 weeks of the intervention, while individuals in the control group lost only 1.1% of their body weight (from 72.98 to 72.19 kg) (Table 2).

No significant interaction was found between the sex hormone profiles and fasting insulin levels, as shown in Table 3. However, a significant group x time interaction was observed in insulin PP2 (*X*^2^ *=* 5.087, *p* = 0.024, φ_c_ = 0.45), with a greater decrease in the lifestyle modification group compared to the control group. Similarly, there were significant changes in hirsutism and depression scores after the intervention between the groups (*X*^2^ *=* 4.910, *p* = 0.027, φ_c_ = 0.25; *X*^2^ *=* 5.553, *p* = 0.018, φ_c_ = 0.42, respectively).

No one reported pregnancy during or after the intervention in either group.

## 4. Discussion

The primary objective of this study was to assess the effectiveness of a 12-week lifestyle modification program using an integrated mobile app designed for overweight women with PCOS. The mobile application had various features, including disease information, diet and exercise therapy, weight management, menstrual period tracking, and questionnaires related to acne, hirsutism, and depressive symptoms [23]. Through the implementation of this evidence-based mobile application, we aimed to address lifestyle modifications required to manage POCS symptoms in overweight women. The results of this study yielded significant findings that enhance our knowledge of symptom management in PCOS and the potential benefits of weight loss, changes in related metabolic indices, and alleviation of depression in overweight women with PCOS.

Weight loss is a primary goal in PCOS management, as it has been shown to improve clinical symptoms [33]. However, achieving and maintaining weight loss can be challenging for women with PCOS because of the lack of personal support, environmental assistance, and professional guidance [34]. To address this gap, mobile applications have emerged as promising tools for treating and preventing obesity and reducing healthcare costs, offering personalized support and accessibility compared with traditional approaches [35]. Nevertheless, the availability of PCOS-related applications for lifestyle modifications, particularly weight management, highlights the need for more expertise and evidence-based content [36]. In our previous study, we developed an integrated mobile application that combines evidence-based information and motivation for lifestyle modifications [23]. The results of this study revealed significant weight loss in the treatment group following the use of the 12-week integrated mobile application, which is consistent with the findings of a previous systematic review that demonstrated the positive effects of lifestyle modification programs on weight and body composition [33]. A weight loss of 5–10% is deemed clinically meaningful and has positive effects on metabolic, reproductive, and psychological health, as stated in the International PCOS Guidelines [37]. However, the weight loss achieved in the present study (4.2% reduction in the treatment group vs. 1.1% reduction in the control group) was not clinically significant. This may explain why there was no significant reduction in fasting insulin levels, which is a commonly used metabolic index. This finding aligns with those of previous systematic reviews emphasizing that weight loss below 5% of body weight may not lead to a reduction in fasting insulin levels [16]. While fasting insulin levels primarily reflect insulin resistance and overall insulin sensitivity, postprandial insulin levels are influenced by factors such as carbohydrate intake and insulin secretion [38]. The dietary controls included in our intervention may have contributed to the regulation of insulin secretion in response to meals.

Moreover, significant improvements in depression were observed in the treatment group compared with the control group. These findings are consistent with those of previous research indicating that weight loss programs for PCOS can lead to enhanced QoL and alleviation of depressive symptoms in overweight women with PCOS [39]. Notably, obesity has a significant impact on depressive symptoms, even in individuals without other clinical symptoms [40,41]. Our study aimed to enhance the participants’ motivation by providing accessible and personalized support through mobile applications or phone calls, which likely contributed to the management of their emotional status and positively influenced the psychological aspects of PCOS. Additionally, regular exercise, which is a part of lifestyle interventions, plays a significant role in alleviating depressive symptoms. Engaging in regular exercise promotes the release of endorphins, which are natural mood-boosting chemicals in the brain [42]. Furthermore, exercise distracts individuals from negative thoughts and promotes a sense of accomplishment and self-confidence [43]. Incorporating exercise as a part of a comprehensive intervention approach could be beneficial for managing depressive symptoms. Lifestyle interventions, such as those implemented in our study using an integrated mobile application, offer a promising avenue for addressing depression in PCOS.

No statistically significant differences were observed in sex hormone levels in the present study. However, it is important to acknowledge that the 12-week duration of the intervention may not have been sufficient to exhibit changes in these hormonal profiles. Although the treatment group showed significant improvements in PCOS-related symptoms, particularly hirsutism and acne, it is important to note that their baseline hirsutism and acne scores were comparatively lower than the general POCS population [27,28], making it challenging to claim the presence of noticeable symptoms. Additionally, although there were significant changes, the observed score changes may not have been clinically significant, making it challenging to determine the effectiveness of the 12-week integrated mobile application for lifestyle modifications. Therefore, further research with longer intervention periods may be necessary to assess the impact of this integrated application on the hormonal profiles in these populations.

Overall, the findings of this study highlight the potential effectiveness of mobile applications as tools to facilitate sustainable weight management in overweight women with PCOS. Although the weight loss achieved was not clinically significant, this study provides valuable insights into symptom management and highlights the potential role of mobile applications in supporting lifestyle modifications in overweight women with PCOS. Further research should explore additional strategies to enhance weight loss outcomes in this population, including the incorporation of advanced technologies such as AI algorithms and the analysis of user lifelogs to provide personalized and tailored dietary guidance. Implementation of an automated smart healthcare system is necessary to raise awareness and guide nutritional intake, thereby improving nutritional management systems. Tracking nutrient intake and recommending appropriate foods to meet the daily nutritional needs of patients have emerged as potential solutions for managing PCOS and improving the QoL of the affected individuals [44]. Additionally, it is important to investigate the long-term effects of these interventions on metabolic and reproductive aspects in larger samples of women with PCOS as well as their impact on overall health and well-being. Furthermore, it is worth noting that the primary limitation of the current study was its relatively small sample size compared with that of previous trials, despite the study’s capability to identify clinically significant differences. These findings have significant implications for healthcare professionals. The potential for integrating mobile applications into PCOS management should be highlighted as this approach can effectively address multiple aspects of the condition and enhance the effectiveness of interventions. By leveraging the convenience and accessibility offered by mobile technology, healthcare professionals can optimize PCOS management and improve patient outcomes.

## 5. Conclusions

The findings of this study highlight the potential benefits of using an integrated mobile application for lifestyle modification in overweight women with PCOS. Significant weight loss, along with improvements in postprandial insulin levels and depression, suggests that such interventions can positively affect the physical and psychological symptoms of individuals with PCOS. Mobile applications have the potential to address the unique challenges faced by women with PCOS by offering personalized support, accessibility, and cost-effectiveness. Further research is warranted to explore the long-term effects and applicability of this intervention.

## Figures and Tables

**Table 1:** General characteristics of participants (n = 28).

	Treatment (Lifestyle Modification) Group (n = 14)	Control Group (n = 14)	*p*
Age, years	28.50 ± 4.83	25.36 ± 4.40	0.153
Height, cm	164.27 ± 5.58	161.11 ± 6.03	0.162
Weight, kg	75.84 ± 9.52	72.98 ± 9.61	0.471
BMI, kg/m^2^	28.53 ± 4.02	27.79 ± 3.05	0.619
LH, lU/L	8.71 ± 3.42	8.48 ± 5.92	0.927
FSH, mlU/ml	5.71 ± 1.43	5.48 ± 1.85	0.989
Estradiol, pg/ml	56.55 ± 34.65	72.91 ± 84.96	0.633
Testosterone, ng/dL	40.55 ± 17.17	40.83 ± 14.05	0.971
SHBG, nmol/L	42.42 ± 33.50	48.31 ± 46.06	0.755
DHEA-S, ug/dL	244.93 ± 107.17	249.60 ± 96.00	0.656
Insulin fasting, μIU/mL	15.28 ± 6.90	16.09 ± 9.92	0.829
Insulin PP2, μIU/mL	87.77 ± 65.33	81.50 ± 52.46	0.732
Hirsutism (0–36)	14.83 ± 2.66	14.83 ± 2.66	0.015
Acnes (0–52)	9.83 ± 8.39	6.40 ± 4.22	0.415
Depression (0–60)	24.15 ± 7.03	16.08 ± 7.63	0.039

Data are summarized as means and standard deviations. Abbreviations: BMI, body mass index; LH, luteinizing hormone; FSH, follicle-stimulating hormone; estradiol; SHBG, sex hormone-binding globulin; DHEA-S, dehydroepiandrosterone sulfate.

**Table 2:** Body mass index for adjusting analysis between the two groups (n = 28).

		Treatment (Lifestyle Modification Group) (n = 14)	Control Group (n = 14)	Group × Time *X*^2^ (*p*)
Weight, kg	Pre	75.84 ± 9.52	72.98 ± 9.61	3.939 (0.042)
	Post	72.65 ± 10.15	72.19 ± 10.37	

**Table 3:** Secondary outcomes after adjusting for hirsutism and depression in the two groups (n = 28).

		Treatment (Lifestyle Modification Group) (n = 14)	Control Group (n = 14)	Group × Time *X*^2^ *(p)*
LH, lU/L	Pre	8.71 ± 3.42	8.48 ± 5.92	0.253 (0.615)
	Post	7.95 ± 3.40	8.67 ± 8.27	
FSH, mlU/ml	Pre	5.71 ± 1.43	5.48 ± 1.85	0.598 (0.439)
	Post	5.70 ± 2.13	5.59 ± 2.04	
Estradiol, pg/ml	Pre	56.55 ± 34.65	72.91 ± 84.96	0.721 (0.593)
	Post	71.73 ± 63.62	70.81 ± 76.32	
Testosterone, ng/dL	Pre	40.55 ± 17.17	40.83 ± 14.05	1.682 (0.195)
	Post	34.26 ± 12.01	39.54 ± 17.41	
SHBG, nmol/L	Pre	42.42 ± 33.50	48.31 ± 46.06	0.101 (0.750)
	Post	57.74 ± 65.20	58.01 ± 54.34	
DHEA-S, ug/dL	Pre	244.93 ± 107.17	249.60 ± 96.00	2.305 (0.129)
	Post	266.20 ± 81.09	291.87 ± 97.26	
Insulin fasting, μIU/mL	Pre	15.28 ± 6.90	16.09 ± 9.92	0.543 (0.461)
	Post	13.22 ± 7.08	14.77 ± 7.90	
Insulin PP2, μIU/mL	Pre	87.77 ± 65.33	81.50 ± 52.46	5.087 (0.024)
	Post	66.20 ± 40.30	72.21 ± 33.56	
Hirsutism (0–36)	Pre	14.83 ± 2.66	11.38 ± 4.61	4.910 (0.027)
	Post	11.85 ± 3.11	11.54 ± 2.95	
Acnes (0–52)	Pre	9.83 ± 8.39	6.40 ± 4.22	2.145 (0.137)
	Post	6.08 ± 6.50	6.07 ± 4.12	
Depression (0–60)	Pre	24.15 ± 7.03	16.08 ± 7.63	5.553 (0.018)
	Post	16.42 ± 13.26	14.38 ± 9.68	

Data are summarized as means and standard deviations. Abbreviations: BMI, body mass index; LH, luteinizing hormone; FSH, follicle-stimulating hormone; SHBG, sex hormone-binding globulin; DHEA-S, dehydroepiandrosterone sulfate.

## Data Availability

The datasets generated in this study are available from the corresponding author upon reasonable request.
